# Googling Food Webs: Can an Eigenvector Measure Species' Importance for Coextinctions?

**DOI:** 10.1371/journal.pcbi.1000494

**Published:** 2009-09-04

**Authors:** Stefano Allesina, Mercedes Pascual

**Affiliations:** 1 National Center for Ecological Analysis and Synthesis, Santa Barbara, California, United States of America; 2 Department of Ecology and Evolutionary Biology, University of Michigan, Ann Arbor, Michigan, United States of America; 3 Santa Fe Institute, Santa Fe, New Mexico, United States of America; 4 Howard Hughes Medical Institute; University of California San Diego, United States of America

## Abstract

A major challenge in ecology is forecasting the effects of species' extinctions, a pressing problem given current human impacts on the planet. Consequences of species losses such as secondary extinctions are difficult to forecast because species are not isolated, but interact instead in a complex network of ecological relationships. Because of their mutual dependence, the loss of a single species can cascade in multiple coextinctions. Here we show that an algorithm adapted from the one Google uses to rank web-pages can order species according to their importance for coextinctions, providing the sequence of losses that results in the fastest collapse of the network. Moreover, we use the algorithm to bridge the gap between qualitative (who eats whom) and quantitative (at what rate) descriptions of food webs. We show that our simple algorithm finds the best possible solution for the problem of assigning importance from the perspective of secondary extinctions in all analyzed networks. Our approach relies on network structure, but applies regardless of the specific dynamical model of species' interactions, because it identifies the subset of coextinctions common to all possible models, those that will happen with certainty given the complete loss of prey of a given predator. Results show that previous measures of importance based on the concept of “hubs” or number of connections, as well as centrality measures, do not identify the most effective extinction sequence. The proposed algorithm provides a basis for further developments in the analysis of extinction risk in ecosystems.

## Author Summary

Predicting the consequences of species' extinction is a crucial problem in ecology. Species are not isolated, but connected to each others in tangled networks of relationships known as food webs. In this work we want to determine which species are critical as they support many other species. The fact that species are not independent, however, makes the problem difficult to solve. Moreover, the number of possible “importance'” rankings for species is too high to allow a solution by enumeration. Here we take a “reverse engineering” approach: we study how we can make biodiversity collapse in the most efficient way in order to investigate which species cause the most damage if removed. We show that adapting the algorithm Google uses for ranking web pages always solves this seemingly intractable problem, finding the most efficient route to collapse. The algorithm works in this sense better than all the others previously proposed and lays the foundation for a complete analysis of extinction risk in ecosystems.

## Introduction

The robustness of ecosystems to species losses is a central question in ecology given the current pace of extinctions and the many species threatened by human impacts [1]–[3]. The loss of species in complex ecological networks can cascade into further extinctions because of the mutual dependence of species. Of all the possible causes leading to these “cascading” extinctions, the simplest case to analyze is that of species left with no exploitable resources [4]–[8]. These extinctions due to lack of nutrient flows represent the most predictable subset of secondary losses and also the best case scenario, since the addition of other effects [9],[10], related to the loss of dynamical regulation, will result in additional losses. The former scenario is the simplest to analyze because the extinction of consumers that are left with no resources will happen with certainty, unless the consumers can switch to a different set of resources. Because modern data sets are obtained by sampling extensively a system over time, it is unlikely that potential resources resulting from switching prey go unregistered. If these potential interactions have been included in the prey of a given predator, then the dynamics of extinction for this flow-based case are completely described by the network structure. This simple analysis also represents the best case scenario, since other causes of extinction such as low population abundance can increase the loss of species in response to the original disturbance, but cannot prevent flow-based extinctions from happening. From the flow-based perspective, the effects of a single species loss can be easily analyzed [7], but those of multiple losses and sequences of extinctions rapidly become an intractable problem.

Species' importance in this context has been traditionally measured using local network properties, such as the number of species' connections [4],[5]. In particular, species with a large number of links are considered keystones (or hubs [11]) for the robustness of ecological networks [5],[6],[8],[12]. A different take on species' importance in networks makes use of centrality measures: species that are central mediate the interaction among those that are more peripheral and therefore should be considered the most important species [13]–[15].

Here we propose a new algorithm for assessing the importance of species for food web robustness that takes into account the full topology of the network. When species importance from the perspective of robustness is correctly measured, the ordered removal of species according to this ranking should lead to the fastest collapse of the network. Our approach inspired by PageRank^TM^, the algorithm at the heart of Google^TM^ [16], uses a recursive definition: a species is important if important species rely on it for their survival. Results show that the algorithm outperforms all other measures of species importance from the perspective of fastest route to collapse. Moreover, it performs as well as a genetic algorithm [17],[18], an evolutionary intensive search that can evaluate millions of solutions, even if the eigenvector implementation is much simpler and faster. A biological interpretation of species importance follows naturally as the amount of matter flowing through a given species, for both qualitative networks constructed from the presence and absence of links, and quantitative networks for which interaction strengths are explicitly specified [19]–[21]. The proposed approach provides the basis for a more comprehensive treatment of extinction risk in food webs.

## Materials and Methods

The World Wide Web is a directed network in which web pages (nodes) are connected with each other by hyper-links. We can write a matrix 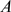 in which the presence and absence of a link from the row-page 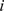 to the column-page 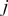 are represented as entries 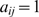 and 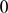, respectively. PageRank^TM^ rates pages as important if they receive links from pages that are in turn also rated as important. The PageRank^TM^ algorithm solves this recursive definition using a clever application of linear algebra [16]. Each page 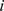 is assigned an importance, and each link 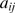 (exiting page 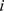 to enter page 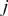) carries an equal fraction of the importance value. The importance of a page is the sum of the importance assigned to the incoming connections. The recursive problem can be solved by building a matrix 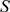 in which each element represents the fraction of importance assigned to a linkand given by 
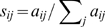
. When matrix 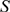 satisfies two conditions (it is both irreducible and primitive [16]), then the problem of assigning importance is solved by computing a fundamental and well-known quantity in linear algebra, the eigenvector 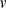 associated with the dominant eigenvalue 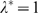 of 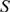. If the two conditions are met, the Perron-Frobenius Theorem guarantees the existence of this dominant eigenvector (Text S1).

One main problem, besides the numerical challenge of computing the eigenvectors of a matrix with several billions rows and columns, is that the World Wide Web is not irreducible [16]. For irreducible matrices, the associated network must be strongly connected, with any two nodes connected by a directed pathway. Because he WWW clearly does not meet this condition, the matrix is modified by applying a “damping factor”, 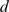. A new matrix 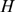 is constructed with entries 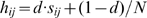, where 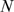 is the number of nodes in the network. The damping factor effectively mimics the probability 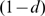 that a user browsing the web can decide to move directly to another (random) page [16]. The eigenvector is then computed for 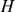.

**Figure 1:**
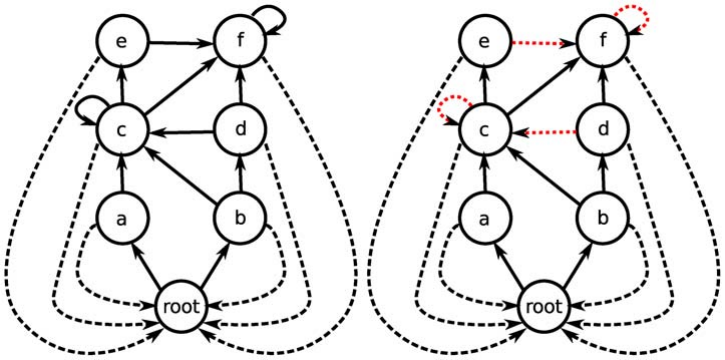
Modification of food webs from ecological considerations to satisfy the two constraints required for application of the *EIG* algorithm. Left) A special node is added to the food web by connecting this “root” to the primary producers. Every species in turn connects to the root to represent the buildup of detritus (dashed line). Right) The analysis can be improved by removing the “redundant” connections that do not contribute to robustness (dashed, in red).

Here we propose an algorithm to rank the importance of species for food web robustness that uses a similar principle. Nutrients move from one species to another in a food web through feeding links. For their survival, species must be able to receive energy and matter from primary producers through some pathway in the network [7],[22]. Thus, we define a species as important if it supports (directly or indirectly) other species that are in turn important. The problem is similar to that of ranking web pages, with the difference that now importance moves in the opposite direction than that of the links (i.e. a web page is important if important pages point to it; species are important if they point to important species). Also food webs are neither irreducible nor primitive, but we can find a biologically sound solution to this problem. A damping factor would be completely unrealistic since nutrients cannot randomly “jump” among links in the food web. We make instead two observations: first, all matter in the food web must originate from primary producers who receive it from the external environment and channel it through the food web to all other species through feeding pathways [21],[23]. We therefore attach to the network a special node (a “root”) that points to all the primary producers [7],[22]. Second, every species has an intrinsic loss of matter which can be represented by adding a link from every node to the root. This process represents the buildup of detritus that in turn is partly recycled into the food web [21],[23]. With these two modifications any food web becomes irreducible and primitive (Fig. 1, Text S1) and we can now solve the problem of assigning importance by computing the eigenvector 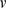 associated with the dominant eigenvalue 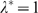. For simplicity, we consider the normalized eigenvector so that 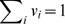.

Recent research on food web robustness has emphasized the role of connectivity: species with a high number of connections are likely to be essential for the survival of other species [4]–[8]. In-silico extinction experiments also showed that random removal sequences rarely cascade in the secondary loss of species, whereas the removal of highly connected species is likely to generate many secondary extinctions. Another line of research borrowed measures of centrality from sociology. Central species mediate the spread of disturbances through the network. In this sense, species with high centrality would be considered “keystone” to the maintenance of connectivity in networks [13]–[15].

To test our algorithm, we performed in-silico extinction experiments in which a single species is removed at each step and the number of secondary extinctions is recorded. We compared several simple algorithms: a) the removal of the most connected species at each step (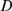, where we measured the number of connections coming out of each node); b) the removal of species according to closeness centrality (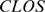): nodes are highly central from this point of view if they have short distance to many nodes in the network; c) the removal according to betweeness centrality (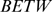): a node has high betweeness if it lies on the shortest path between many couples of nodes; d) removal according to dominators (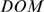): a 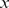 node dominates another 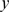 if all the paths from “root” to 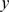 contain 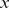 - the removal of 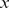 will therefore drive 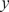 extinct [7]; finally, e) we removed according to the eigenvector-based algorithm outlined above (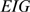).

**Figure 2:**
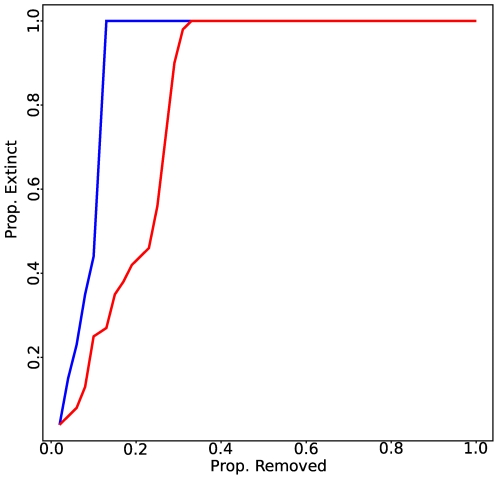
The extinction area is the area described by the area below the curves. The area can take values from 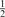 (no secondary extinctions in response to the removal of species) to 1 (all species go extinct after the first removal). The 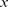 axis represents the fraction of species removed in the numerical experiment, while the 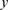 axis is the fraction of species that are extinct as the result of these removals. The example uses the St. Mark's food web [29] and the 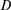 (red) and 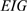 (blue) algorithm.

All the algorithms are “greedy”: at each step, we compute the “importance” of each node according to a particular algorithm, and we remove the one with the highest importance. The procedure is repeated until all the species have gone extinct or have been removed. The algorithms are explained in detail in the Text S1. For each extinction sequence, we measured the “extinction area”, a quantity that equals 1 when all species go extinct after the first removal and tends to 1/2 when no secondary extinction is observed (Fig. 2). In this way, we can assess the performance of each algorithm with a single number. If important species are removed early on, then the area will be larger.

The algorithms could yield ties - nodes with the same importance. Whenever we encountered ties, we considered all the possible sequences of extintions that may result exploring all the ties. Therefore, algorithms with low ranking power (i.e. yielding many ties) could produce very many extinction sequences. We followed all extinction sequences generated by ties whenever they were less than half a million. If there were more possible solutions, we analyzed the first half million.

We applied all the algorithms to 12 published food webs (Table 1). For each algorithm and network, we tracked the total number of solutions produced by the algorithm, the minimum, maximum and mean “extinction area” and the number of solutions yielding the maximum area (Text S1).

**Table 1:** Extinction Area.

Food Web	Num. Species	Max. D	Max. CLOS	Max. BETW	Max. DOM	Max. EIG1	Max. EIG2	GA	Reference
benguela	29	0.7943	0.7539	0.9025	0.9798	0.9798	0.9798	0.9798	[31]
bridge	25	0.6160	0.7888	0.8384	0.5904	0.8384	0.8384	0.8384	[32]
chesapeake	31	0.8949	0.8273	0.8241	0.8720	0.9251	0.9251	0.9251	[33]
coachella	29	0.8288	0.7979	0.8811	0.9394	0.9394	0.9394	0.9394	[34]
grass	61	0.8995	0.8866	0.8804	0.9481	0.9481	0.9481	0.9481	[35]
reef	50	0.7632	0.7180	0.7700	0.9640	0.9640	0.9640	0.9640	[36]
shelf	79	0.6380	0.6561	0.8103	0.9885	0.9885	0.9885	0.9885	[37]
skipwith	25	0.6560	0.6448	0.6448	1.0000	1.0000	1.0000	1.0000	[30]
stmarks	48	0.8550	0.6263	0.6680	0.9180	0.9197	0.9210	0.9210	[29]
stmartin	42	0.8067	0.8050	0.8571	0.9036	0.9178	0.9178	0.9178	[38]
ythan91	83	0.9505	0.9205	0.9554	0.9772	0.9772	0.9772	0.9772	[39]
ythan96	124	0.9349	0.9448	0.9685	0.9781	0.9807	0.9807	0.9807	[40]

**Figure 3:**
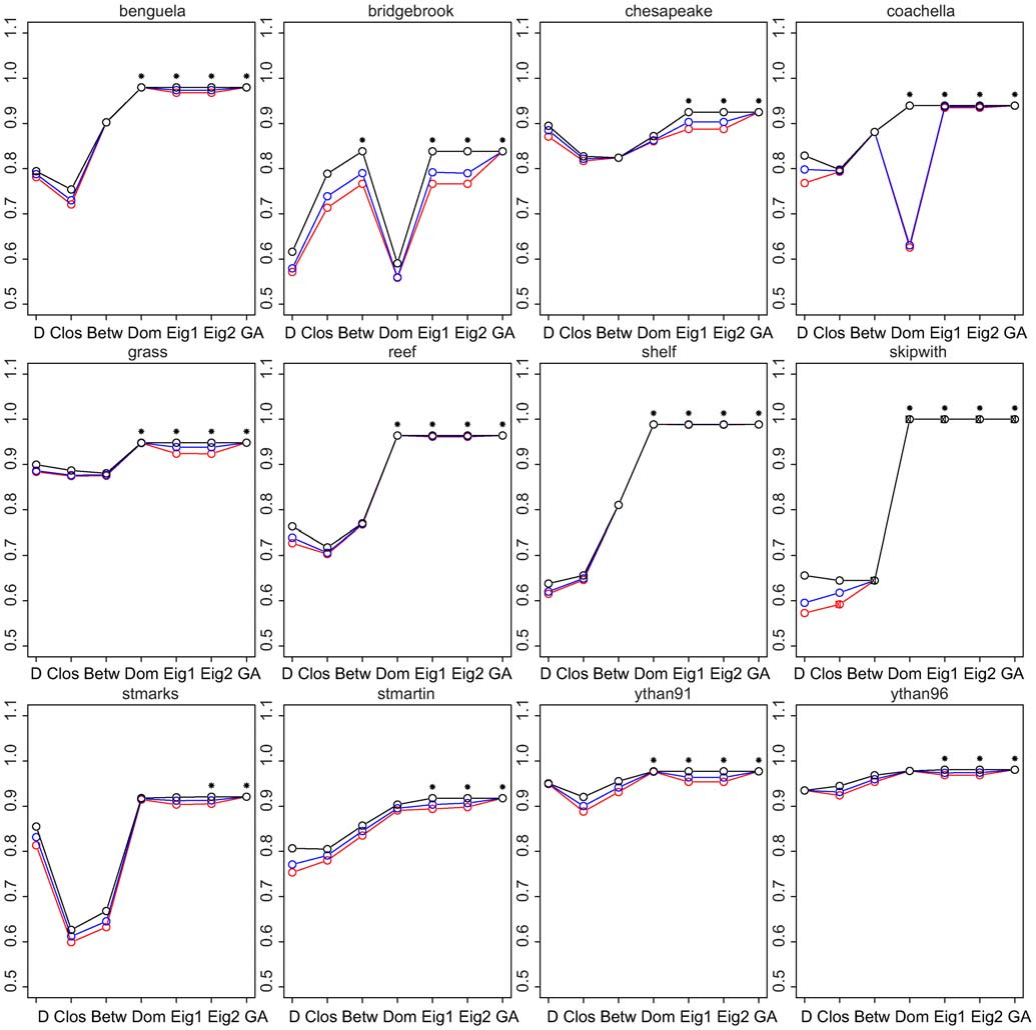
Extinction areas for 12 published food webs (Table 1) according to the 7 algorithms presented in the text. The area is 1 (as in the “skipwith” [30] food webs) only when there is a single primary producer. Because each algorithm can give raise to several solutions, we report the minimum (red), mean (blue) and maximum (black) registered extinction area. We indicate with an asterisk “*” the algorithms that are able to match the performance of the genetic algorithm (GA).

For each food web, we report the number of nodes, and the maximum “extinction area” (Fig. 2) obtained using the algorithms presented in the text (Fig. 3).

We then evaluated the value of the maximal extinction area. Because the number of possible removal sequences is 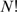 where 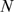 is the number of species in the network, the enumeration of all possible cases is clearly unfeasible. We therefore programmed a Genetic Algorithm [17] (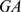) that seeks to find the best possible sequence using an evolutionary search. This type of algorithm has been shown to be effective for similar problems in food web theory [18], even when computationally expensive and when its performance declines with food web size. Here, the 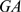 search performs at least as well as the best among the other algorithms, as expected for an effective search (Fig. 3).

## Results

In all cases, the best solution for the degree-based algorithm (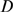) and the closeness centrality (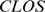) did not match the genetic algorithm (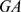): these measures do not correctly identify the fastest route to collapse (Fig. 3). Betweeness centrality yields an area as large as that of the 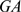 in only 1 case (*benguela*). The dominators-based procedure finds the best solution in 2/3 of the cases. The eigenvector-based algorithm finds the best solution in 11 cases out of 12. To improve the 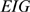 algorithm, we build upon a previous approach of ours [22], based on the observation that not all the links in a food web contribute to robustness. The idea that more complex networks would contain a multiplicity of pathways that would in turn render the networks more robust was put forward by MacArthur more than fifty years ago [24]. We recently showed that, while this is generally true, some links do not contribute to robustness, while others dampen the effects of species removal and increase robustness (Fig. 1) [22]. Thus links can be classified as “redundant” or “functional” from the perspective of their effects on secondary extinctions. From this classification, one can obtain a simplified food web by removing all redundant connections, that has exactly the same robustness properties than the original network in terms of the secondary extinctions. For the algorithm 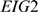, then, we repeated the removal sequence experiment but we computed 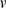 for the simplified food web obtained by first removing redundant connections. The results indicate that the algorithm is capable of finding the best solution provided by the 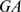 in all cases (Fig. 3, Text S1).

## Discussion

We have developed two algorithms to rank species in food webs according to their role in extinction cascades. We considered a flow-based perspective in which species go extinct if they lack a connection through some pathways to primary producers. Although it is evident that many other types of extinctions can increase total species loss, the subset considered here provides a baseline and corresponds to the best case scenario in which the minimum impact to the network is taken into account. Species left with no resources will go extinct, unless they can switch their choice of prey sufficiently fast. It is known that species can exhibit this type of adaptive behavior in response to the relative abundance of prey, with consequences for the stability of predator-prey systems [25]. Because the food webs we have analyzed are sampled in the field over time and space, it is most likely that the links included in the networks already reflect prey switching. An important source of additional secondary extinctions will be related to the population dynamics of species. The complete consideration of dynamics with a system of nonlinear differential equations that simulates the outcome of species losses, will only increase the number of species predicted to go extinct by the simplest scenario. The analysis of removal effects remains very challenging if not prohibitive for large ecological networks (but see [9],[10],[26]), requiring information most often unavailable on the functional form of a large number of interactions and their associated parameters, the exploration of different assumptions and a huge parameter space. The simple and elegant solution for the flow-based case provides a baseline from which additional impacts can be considered.

The results obtained here with a simple algorithm emphasize that the position of a species in the food web, rather than its sheer number of connections, is the main determinant of its impact on extinction cascades. This contrasts with the emphasis given so far to the number of connections and to the concept of “hubs” in networks. We have shown that the performance of the 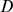 algorithm, which considers only the neighbors of a given species, is considerably worse than that of the eigenvector based algorithms at finding the fastest route to collapse. The latter algorithms solve the problem of importance by considering the full topology of the network and the particular position that each species occupies. We further showed that an algorithm that first removes “redundant” connections provides a valuable improvement, because it relies on the functional role of connections in maintaining the flow of nutrients through the food web. Interestingly, a parallel problem has been analyzed in computer science (Text S1).

Srinivasan *et al.* [27] have shown that many realistic removal sequences are not likely to cascade in massive species' losses, with the loss of threatened species not necessarily resulting in further extinctions. It is therefore difficult to discriminate importance among species whose removal has little direct effect on network structure. The eigenvector approach provides a simple and effective way of comparing species importance even when their removal does not result in extinction cascades. This should help assessing the relative importance of threatened species for network robustness and from the perspective of network structure. Coll *et al.* analyzed the effect of actual human-induced extinction in the Mediterranean sea and found that removing commercially valuable species had typically a higher impact than random removals, but lower than maximum degree driven removals [28].

The dominant eigenvector has also a simple biological interpretation. To show this, we assume for the moment that we can fully describe the interacting community by means of differential equations representing the dynamics of species' abundance, 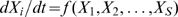. We further consider that the system is at a feasible equilibrium point (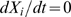 for all species, 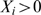). For this case, we can measure the flow of biomass entering and exiting each species (for example, in kilograms of biomass per year per hectare) and the amount entering and exiting each node must be equal given the equilibrium condition [19]–[21]. These quantities are proportional to the eigenvector used here: specifically, 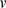 provides an estimate of the flow through each species (Text S1, Fig. S1). In the absence of available information on diet preferences, 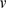 measures the flow that each species would receive if each of its prey provided equal amounts of nutrients. When quantitative information on these inputs is available, 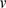 and the flow-based description become exactly equivalent (Text S1, Fig. S2).

The proposed algorithm further provides a measure of eigenvector centrality in directed, rooted networks. Other centrality measures have been proposed to evaluate species importance [13]–[15], but they typically consider undirected networks and have not been adapted to food webs. This is reflected in the poor performance achieved by the centrality algorithms. Here we have shown that consideration of ecological knowledge on food web processes can improve algorithms that have been developed in other branches of science. It should be possible to adapt the methods presented here to other types of biological networks, especially metabolic ones. For food webs, the next challenge is to add other dynamical effects to this framework, to obtain a more complete description of extinction risk in complex ecological networks.

## Supporting Information

Text S1 Supporting Information
(0.03 MB TEX)

Figure S1 The Lovinkhoeve Experimental Farm food web modified as described in the text. The flows are expressed in kg of biomass per year per hectare. Red links represent biomass losses experienced by the species.
(1.50 MB EPS)

Figure S2 Relationship between the size of flows in the food web (Fig. S1) and the values of the eigenvalue vi. The *y* axis is the sum of all flows entering (or exiting) a species. The *x* axis is the corresponding value in the eigenvector *v*. The logarithms of both values are shown in the graph to better discriminate the points.
(0.50 MB EPS)
